# Synergizing expectation and execution for stroke communities of practice innovations

**DOI:** 10.1186/1748-5908-5-44

**Published:** 2010-06-08

**Authors:** Lise Poissant, Sara Ahmed, Richard J Riopelle, Annie Rochette, Hélène Lefebvre, Deborah Radcliffe-Branch

**Affiliations:** 1 Centre for Interdisciplinary Research in Rehabilitation of Greater Montreal, Montreal, Quebec, Canada; 2 Institute of Rehabilitation Gingras-Lindsay of Montreal, Montreal, Quebec, Canada; 3 School of Rehabilitation, University of Montreal, Montreal, Quebec, Canada; 4 School of Physical and Occupational Therapy, McGill University, Montreal, Quebec, Canada; 5 Neurology and Neurosurgery Department, McGill University, Montreal, Quebec, Canada; 6 Faculty of Nursing, University of Montreal, Montreal, Quebec, Canada

## Abstract

**Background:**

Regional networks have been recognized as an interesting model to support interdisciplinary and inter-organizational interactions that lead to meaningful care improvements. Existing communities of practice within the a regional network, the Montreal Stroke Network (MSN) offers a compelling structure to better manage the exponential growth of knowledge and to support care providers to better manage the complex cases they must deal with in their practices. This research project proposes to examine internal and external factors that influence individual and organisational readiness to adopt national stroke best practices and to assess the impact of an e-collaborative platform in facilitating knowledge translation activities.

**Methods:**

We will develop an e-collaborative platform that will include various social networking and collaborative tools. We propose to create online brainstorming sessions ('jams') around each best practice recommendation. Jam postings will be analysed to identify emergent themes. Syntheses of these analyses will be provided to members to help them identify priority areas for practice change. Discussions will be moderated by clinical leaders, whose role will be to accelerate crystallizing of ideas around 'how to' implement selected best practices. All clinicians (~200) involved in stroke care among the MSN will be asked to participate. Activities during face-to-face meetings and on the e-collaborative platform will be documented. Content analysis of all activities will be performed using an observation grid that will use as outcome indicators key elements of communities of practice and of the knowledge creation cycle developed by Nonaka. Semi-structured interviews will be conducted among users of the e-collaborative platform to collect information on variables of the knowledge-to-action framework. All participants will be asked to complete three questionnaires: the typology questionnaire, which classifies individuals into one of four mutually exclusive categories of information seeking; the e-health state of readiness, which covers ten domains of the readiness to change; and a community of practice evaluation survey.

**Summary:**

This project is expected to enhance our understanding of collaborative work across disciplines and organisations in accelerating implementation of best practices along the continuum of care, and how e-technologies influence access, sharing, creation, and application of knowledge.

## Background

Each year, over 50,000 Canadians suffer a stroke [1,2]. With improved awareness and with enhanced system responses, specialized diagnostics and therapeutic procedures, a large majority of individuals now survive their stroke. With declining stroke-related mortality rates and the aging population, stroke will become a highly prevalent condition [1] and will have great impact on the use of healthcare resources [3]. To offset this demand-side convergence situation requires an appropriate use of existing resources and the development of new supply-side resources that can answer patients' needs more effectively and efficiently. Recently, post-hospital care went through a major reorganisation of services, concentrating inpatient stroke rehabilitation in specific rehabilitation hospitals and implementing a centralised referral process for rehabilitation care [4]. Concurrently, emerging structures for services delivery in Quebec, namely the local health networks and the health and social services centres (CSSS) offer a unique opportunity to implement innovative models of care delivery. Validated models that build upon continuity of services and care efficiently and effectively can optimize chronic disease management and control costs for populations with specific needs [5,6], including stroke. Creating stroke care continuums represent highly relevant solutions to deal with predictors of discontinuous care--involvement of multiple care providers from different disciplines and organisations [7,8].

### The Montreal stroke network

The desire to address informational, management, and relationships gaps between the different care providers involved in stroke care delivery to optimize continuity of care [9] led to the creation of a stroke working group (SWG) in 2005. Over the past five years, the SWG has brought together various stakeholders, including patients, caregivers, clinicians, managers, voluntary organisations (Heart and Stroke Quebec), and researchers; it has developed several projects in the areas of pre-hospital services, acute care, intensive functional rehabilitation, social reintegration, and prevention. Funding from the Canadian Institute of Health Research (Poissant *et al*., 2006 to 2007) allowed the working group to expand to an informal network, the Montreal stroke network (MSN) and to encourage emergent intentional communities of practice (CoPs) (Poissant L, Riopelle R, Rochette A, Boucher J, Alfonso M, Cox N, unpublished comminication).

Today, the MSN comprises over 40 individuals representing the various stakeholder groups from over 15 healthcare organisations associated with one of the two large University health networks--from the University of Montreal and from McGill University. In addition to clinicians, four researchers (LP, AR, SA, DRB), a patient, a caregiver, and a representative from Heart and Stroke Quebec are active members of the MSN. The great majority of members are active participants in one of the four CoPs (acute care, prevention/education, community reintegration, functional rehabilitation). Members have the opportunity to meet face to face on a monthly basis to advance the project of respective CoP; otherwise communications are email-based.

**Figure 1:**
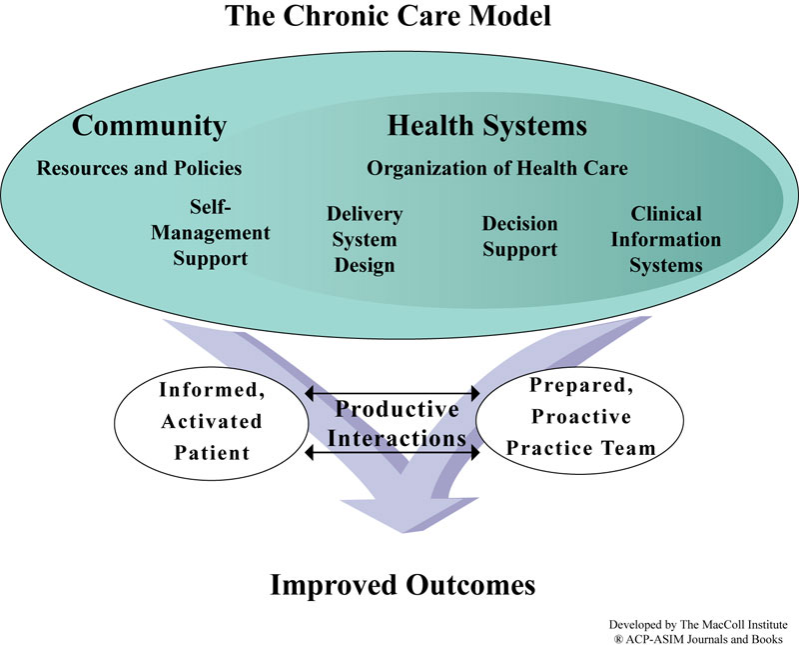
**The chronic disease management model**.

An annual meeting offers members of the MSN a chance to socialize, discuss, and identify priority areas that should be addressed to improve stroke care across the continuum, while providing an opportunity to disseminate and exchange knowledge with clinicians and managers who deliver stroke care, but are yet to be involved in one of the MSN-CoPs. An annual meeting of the MSN reunited over 65 individuals, including policy and decision-makers from the Quebec Health and Social Services Ministry, the Montreal health and Social Services Agency, and from the Heart and Stroke Foundation of Quebec. Participants made a decision to revise their structure to promote the development of concerted activities across the continuum, from prevention to social participation, with the overall objective to better meet the needs of community-dwelling stroke survivors. To that end, the chronic disease management (CDM) model [10] (Figure 1) was chosen by MSN members as a comprehensive framework upon which future activities and projects could be developed.

### The chronic disease management model

Over the past years, chronic care has received a great deal of attention. While the CDM model was developed for chronic diseases such as asthma, hypertension, or diabetes, its elements represent a highly relevant foundation piece upon which to build activities to address the complexity of stroke as a disease, as a disability with implications across the life course for both patients and their families, and as a surrogate for other chronic neurological disorders. The model highlights six interactive elements: the community, the healthcare system, self-management education and support, health services organisations, decision tools, and clinical information systems. Studies have shown that the implementation of different elements of the model can have positive impact on population health [11,12]. The model is particularly interesting to the area of health behaviour/promotion research, as it reinforces the need to inform patients and have them play an active role in their care delivery. It also emphasizes the need for clinical teams to have access to all necessary tools and information for evidence-based health services delivery. At another level, the model underpins the need to put in place dynamic partnerships between the community and the healthcare system on one end and between patients and health professionals on the other end. These partnerships are expected to improve patient's outcomes and improve efficiency and effectiveness of health services delivery [13] through activities in the domain of implementation research which must synergize with the other scientific foundation pieces underpinning innovation in health--clinical, health behavior/promotion, and health services [14].

### CoPs within the MSN

CoPs are interesting structures to facilitate intra- and inter-disciplinary collaborations necessary to accelerate the implementation of the CDM model and of best practices recommendations. They encourage synergy between expectation and execution, support integrated research endeavor in the four scientific foundation pieces of innovation in health, and impose action within a context that reaches every participant's needs. According to Wenger [15], CoPs form the basis of learning organizations. Through mutual engagement and negotiation, CoP participants identify, develop, and finalize a common project. The project can take different forms, from the creation of documents to the application of novel practices [15,16].

To date, emergent CoPs within the MSN have been successful in developing and implementing critical outputs, such as a referral tool that accelerated patients' transition between acute care to rehab [17]. The referral tool combined research and clinical expertise. The work accomplished by one MSN-CoP translated to and was acknowledged by the Montreal Health and Social Service Agency who integrated its content into their provincial referral system providing incredible reinforcement to pursue collaborative work around stroke care delivery. Another CoP reuniting clinicians from two different university health networks led to the development of a bilingual training session that translated to a program offered to over 120 nurses. By sharing their 'know-how' clinicians have successfully impacted upon clinical practice. As a group, participants appreciated the knowledge sharing and expertise access the CoP provides as well as the increased collaboration, problem-solving capacity, and trust. At the organisational level, operational efficiency was an important benefit, along with cost savings and improved service delivery [18]. Improved 'know-how' and capacity to understand and implement best practices are among the benefits most valued by CoP participants [18,19].

There is now a state-of-readiness to leverage on the mutual engagement and accountability that MSN members have developed over the last few years through CoP projects. These will accelerate the development of activities framed by the key elements of the CDM model guided by recommendations forthcoming from the Quebec provincial stroke strategy, and led by Heart and Stroke Quebec, to adopt national stroke best practices, and translate them to the practice environment for uptake and application.

### Need for knowledge translation (KT) activities in stroke care and services

Over the past years, initiatives such as the StrokEngine [20], the stroke evidence-based review [20,21], have been made available to clinicians, managers, patients, and families to improve stroke care. Stakeholders can now more easily access to the most up-to-date knowledge in the area of stroke care. However, access to knowledge, by itself, is unlikely to translate into behavior or practice change [22] unless integrated programs of research as described in this proposal, critical for innovation, are embraced.

Because of the general lack of explicit recognition of the need for the integrative research referred to, to date, very little standardization or systematization with respect to approaches to stroke care exists. As examples: screening for community re-integration or vocational problems prior to discharge or as part of a systematic follow-up of patients, whether they are discharged home from the acute care or rehabilitation hospital, has been identified as best practice [23], but is not widely implemented; community-based follow-up of individuals with stroke has been shown to optimize continuity of care [24] and could potentially prevent the development of handicaps in this high-risk population [25] in large part due to recurrent stroke, the risk of which is increased by a previous stroke; few organizations or teams have routine follow-up assessment of their patients and no standardized tools are in place for assessment and referral to re-integration focused rehabilitation centers. The development and implementation of such tools is expected to reduce care disparities and enhance health-related quality of life (HRQL) of community-dwelling persons with stroke.

In 2006, the Canadian Best Practice Recommendations for Stroke Care emerged from a working group of the Canadian Stroke Strategy [26]. These recommendations (a total of 24) call for an integrated approach to implement best practice that spans prevention, acute treatment, rehabilitation, and recovery in the community. Several provinces, including Quebec, have recently endorsed these recommendations. The Quebec Stroke Strategy (QSS) is led by Heart and Stroke Quebec. Viewed as a successful and affordable model of care delivery by the Quebec MSSS advisory committee for validation of recommendations, the MSN is a natural environment to put into action the QSS through CoPs activities. The ongoing participation of Heart and Stroke Quebec within the MSN will facilitate timely, bi-directional communications between MSN members (clients, clinicians, investigators), decision- and policy-makers.

### Objectives

This research project proposes to examine how MSN members working within CoPs will leverage on translational progress to date, the CDM framework, and upcoming stroke best practices to mobilize knowledge towards developing and implementing innovative, evidence-informed projects throughout the stroke care continuum using the integrated research thrust necessary for health innovation. More specifically, our study, building on a solid portfolio of clinical, health behaviour/promotion, and health services research in the stroke area referred to above, will: examine internal and external factors that influence individual and organisational readiness to change using the methodology of implementation research--a logic model of eight critical success factors predicting the likelihood of change [27]; examine processes used by CoP members to meet individual and organizational expectations while respecting best practice recommendations and the CDM framework; examine the impact of the e-collaborative platform in facilitating KT activities for active CoP participants and peripheral MSN members; and assess user's perception of usefulness of the e-collaborative platform.

## Methods

### Participants

Newly recruited and current organisations (acute care, rehabilitation hospitals, rehabilitation centers, and health and social services centres) and individual members (patients, caregivers, researchers, decision makers, and policy makers) of the MSN will be asked to participate. So far, MSN activities were developed over face-to-face meetings within CoPs, restricting participation of only one to two stroke clinicians per organisation. In this project, we plan to invite all clinicians involved in stroke care among MSN's organisations. From previous work, we estimate to invite over 200 clinicians (nurses, occupational therapists, physical therapists, social workers, speech language pathologists, educators, physicians, neurologists, and psychologists) working with the stroke population across the continuum in the Montreal area. We expect that 40 to 50 participants will join or form CoPs, while others will stay in the periphery as MSN members.

### Implementation plan

#### Development of the e-collaborative platform

E-collaborative platforms that encourage social networking among patients and/or health providers are central to Web 2.0 innovations [28,29]. These platforms are performant tools to share information in a dynamic, bi-directional way [30-32], and to foster innovations https://www.collaborationjam.com. In parallel, e-platforms that are developed to meet the needs of all CoP participants can be useful to support communications and facilitate knowledge sharing between participants [33]. These platforms offer a wide array of functionalities, such as automated wikis (encyclopedia), discussion forums, postings, and direct access to selected scientific papers. Platforms are effective means to create rich, shared repertoires of resources that can be accessed at any time by all members, whether they play an active role or stay in the periphery.

In an era of limited resources and increasing caseloads, attending face-to-face meetings in organizations located only a few kilometers away is perceived as time consuming. So far, MSN members have been respectful of their commitment to their CoP, an essential element to sustainable, evolutive CoPs [34]. However, with the expansion of the MSN, its leading role as a model to successfully move knowledge into action demands additional structures to support communication processes and knowledge exchange. Complementing and extending the face-to-face activities of the CoPs and their communications that have proven to be successful, an e-collaborative platform will be developed to enhance knowledge capture and evolution around members interactions on practice changes to be implemented. An operational committee (researcher, user, and programmer) will be responsible for the development and iterative evaluation process of the platform.

We propose to create our own e-collaborative platform, the Stroke E-Collaborative Interface (SECI) (which also refers to the four phases of Nonaka's knowledge creation model [35]: socialization, externalization, combination, internalization). The technical implementation of SECI will rely, as much as possible, on proven, dedicated tools already available in the form of commercial or open source software. Most likely, the entry website will be presented in the form of a blog or a social network in order to facilitate access to all the information related to a given subject of interest and to encourage comments by visitors. Because interactivity, collaboration, and knowledge transfer are of prime importance, the platform will also offer a dedicated forum, RSS, as well as few collaborative tools needed for brainstorming or to gather opinions (quick surveys) on specific subjects. Access to SECI will be free of charge but will be reserved to registered and invited visitors for the time of the study. This restricted, personalized access will allow implementation of automated alerts via e-mails to all registered users who will select this service. At the beginning of our project, all participants will receive automated alerts as we display in the posting section and discussion forums, the Quebec best practices recommendations in the context of the CDM model.

#### Creating 'jams' around best practices recommendations

In 2003, IBM created its first world 'jam' or online brainstorming session reuniting thousands of experts [36] around a specific problem or question. A restrictive time window (72 hours) is provided to participants, creating a real 'jam' both in terms of volume of communications and in terms of content diversity. During 'jam' sessions, participants have access to everyone's posts to enrich their comment or reaction. The abundant volume of postings is then analysed (IBM uses data mining), and the most feasible solutions are implemented. On-line 'jams' have gained popularity among large private companies who see in this technique a rapid access to innovative solutions [36].

We propose to create 'jams' around each best practice recommendations. Stroke best practice recommendations will be presented one at a time, every second week, on the blog section of the SECI. A 72-hour time window will be provided to participants to engage in an integrative research activity involving clinical research (best practices) and implementation research (using the process logic model) to react, comment, and post solutions around implementation of that best practice recommendation. Only best practices that are relevant to the core group of MSN members (*e.g*., patient/family education, dysphagia assessment, *et al*.) will be presented (approx. 10 to 24). Senior MSN members who have been leading MSN-CoP activities will be invited to be moderators. The research team will not participate in any of the jams to minimize influence over the choice of a solution. Closed jams will remain available for viewing only and become archive documents.

#### From jams to emergent CoPs

Jam postings will be analysed by a member of the research team, within two weeks after each jam closing. Qualitative analyses techniques will be used to identify emergent themes Syntheses of these analyses will be provided to members to help them identify priority areas for practice change, and encourage the creation of new communities of practice. Feedback will be linked to the CDM model (*e.g*., a proposition to adopt across organisation tool × would be linked to the CDM element pertaining to information systems) and brought back to the SECI in the blog area. Members of the research team will turn into active participants in these new threads of discussion fostering interactions aimed at reducing the 'know-do' gap, the primary outcome of KT activities [37]. Discussions will be moderated by clinical leaders already identified from their ongoing work and involvement within the MSN. The moderators' role will be to accelerate crystallizing of ideas around 'how to' implement selected best practices.

#### From CoP to practice change

Given the time frame of this study, we expect that existing CoPs will engage in implementation projects that will lead to practice changes, and that newly created CoPs will identify their respective project and determine the expected deliverable. CoPs will be provided dedicated space on the SECI to facilitate asynchronous (non real-time) communication and facilitate knowledge sharing. CoPs will be encouraged to maintain face-to-face meetings. CoPs leader/moderator will be responsible to set and maintain the rhythm of activities to reach set objectives [38,39], and to ensure active and peripheral participation as necessary is essential to the survival of any CoP.

#### Ethics

The study was approved by the ethics board of the Centre for Interdisciplinary Research in Rehabilitation of Greater Montreal (CER-440-0709).

#### Evaluation plan

Qualitative and quantitative approaches will be used to measure the study objectives.

#### Observations

Activities of CoP members and peripheral members during face-to-face meetings and on the SECI platform will be documented. Content analysis of all activities will be performed using an observation grid that will include key elements of CoPs and of the different phases of the knowledge creation cycle developed by Nonaka. Key variables and outcome indicators are:

1. Mutual engagement: interactions, exchanges on the web-based forum, attendance at meetings, respectful negotiation, attainment of consensus.

2. Common project: identification of a care need to be prioritized, discussion and negotiation towards identification of a common project, operationalization of the common project (goals, steps, resources required, timeline), initiation of the project.

3. Shared repertoire: use of shared information/knowledge in problem-solving strategies, utilization of e-technologies to access and capture information/knowledge, display of explicit and tacit knowledge.

4. Socialization: trust building, active participation, verbal or written communications, development of shared perspectives, sharing of anecdotes, stories, tacit knowledge, common state-of-readiness for mutual engagement.

5. Externalization: development of explicit contributing knowledge, identification of knowledge needs.

6. Combination: systematizing of knowledge, validation and relevance of information shared.

7. Internalization: evidence of learning by doing (appropriation) as manifested by implementation measures.

8. Partnerships: display of mutual aid, shared problem solving, group cohesion, interdisciplinary interactions, mixed group interactions, bidirectional interactions/communications.

#### Individual interviews

Members of an existing MSN-CoP (5 to 10 individuals) and a newly created one (5 to 10 individuals) will be asked to participate in semi-structured interviews. Interviews are expected to last one hour and will be conducted by a graduate student with training in qualitative research. Interviews will be used to collect information on the variables of the KT framework including the Knowledge to Action framework that represents the activities needed for knowledge application [28]. Using information derived from interviews, a logic model of critical success factors predicting the likelihood of change will be developed and will include an assessment of the following components: problem identification; knowledge identification, review, and selection; knowledge adaptation to local context; assessment of barriers to utilization; selecting, tailoring, and implementing knowledge to produce change; monitoring knowledge use; evaluating outcomes; and sustaining ongoing knowledge use. The value-added of the CoP, as well as the barriers and facilitators to the utilisation of SECI and to the implementation of best practices in line with the CDM model, will also be evaluated (objectives 2,3,4). NVivo software will be used to categorize, code, and analyse the information. Interviews will be audiotaped and transcribed verbatim, and written consent of the participants will be obtained at the beginning of the project. Information will be coded to identify emergent themes and concepts.

#### Questionnaires

All participants will be asked, at time of entry in the study, to complete three questionnaires that will give us the capacity to examine individual and organizational characteristics in relation to readiness to change (objective one). The typology questionnaire [40], a 17-item questionnaire that classifies individuals into one of four mutually exclusive categories of information seeking: 'seekers' are typically information-oriented, seeking data from reliable sources and evaluating the information themselves, and altering practice when such evidence warrants a change; 'receptives,' while also information seeking, generally rely on the judgments of respected colleagues and/or incorporate new practices only when they believe there is sufficient evidence; 'traditionalists' believe that experience and authority are the basis on which to make practice decisions; 'pragmatists' tend to focus on the day-to-day practice demands and make practice decisions based on their impact on the efficiency of their practice.

The e-health state of readiness questionnaire [41] is a 58-item questionnaire that covers ten domains (change, care delivery, work processes, personal commitment, skills/knowledge, leadership, communication, support, beliefs about technology, resources and technology) organized under three subscales (individual, organizational, and technological).

A CoP evaluation survey based on an existing evaluation grid [42] will be developed. The CEFRIO grid, in its current form, comprises 45 items, and covers several domains (relationships, collaborations, members' satisfaction, vitality of exchanges, gains to the community, *et al*.) that contribute to the assessment of a CoPs success. Overall, completion of all questionnaires at time of entry in the study should take approximately 30 to 40 minutes per respondents. Questionnaires will be available online on SECI.

#### Expected impact

Over the years, regional networks have been recognized as an interesting model to support interdisciplinary and interorganizational interactions that lead to meaningful care improvements. CoPs activities of the MSN offer a compelling structure to better manage the exponential growth of knowledge and to support care providers to better manage the complex cases they must deal with in their practices. CoPs can benefit the individuals, the community, and the organizations.

Our work with the MSN rehabilitation CoP identified improved continuity of care, effectiveness of care, and collaboration between care providers and organizations as additional benefits of CoPs [17]. Building on solid foundations and a valid framework, this project will allow us to expand our KT activities to a larger group of clinicians combining individuals with various CoP experience, different leadership styles, and different expertise to develop and implement innovative approaches to accelerate evidence-based practice and implementation of the CDM model. Our study will also increase our understanding of how interdisciplinary and interorganizational CoPs can operationalize the elements of the CDM model in the context of stroke management across the continuum of care, and will increase our knowledge on the role of e-technologies in supporting social networking and KT activities in the context of CoP.

This project builds on the existing MSN that has been successful in building human and knowledge capacity through the use of stroke guidelines. The network provides a learning environment, facilitates professional development, and attracts research interest. Through participation of MSN members in strategic committees, the QSS (QSS), Agence's committees, the MSN also has a measure of influence at the policy level in the province of Quebec. Through this project, we hope to reach a larger community of stakeholders throughout Quebec to engage them through concrete projects in the implementation of best practice recommendations for optimal stroke care.

Although this project is a Montreal initiative and is developed around stroke care, we strongly believe the study results will be applicable to other chronic diseases that require management over the care continuum and across personnel and organizations. The MSN is a robust pilot site as the teams deal with both disease and disability through the lifecourse. This provides a platform useful to most other chronic conditions because identical issues exist with respect to dealing with chronic disease leading to disability. In addition, the CoP structure that is being used gives leadership and ownership to the team members, a project design that could be replicated in other chronic disease environments to improve integrated chronic disease management. The proposed research could inform recommendations and, importantly, policy centered on creating successful platforms for integrated chronic disease management.

The MSN is the inter-organizational foundation piece for innovations in stroke that is unique in Canada and promotes developments for a specific demographic--that of Montreal and surrounding areas in the province of Quebec. Understanding factors that influence the development of successful CoP within an interorganisational and interdisciplinary network and assessing the usefulness of e-collaborative platforms will significantly improve the healthcare system's capacity to implement innovative approaches for effective practice changes.

## Competing interests

The authors declare that they have no competing interests.

## Authors' contributions

LP drafted the manuscript and conceived of the study. SA helped draft the manuscript and the study. AR, RR, HL, and DRB critically revised the study and manuscript. All authors read and approved the final manuscript.
